# Dissecting the Molecular Mechanism of 10-HDA Biosynthesis: Role of Acyl-CoA Delta(11) Desaturase and Transcriptional Regulators in Honeybee Mandibular Glands

**DOI:** 10.3390/insects16060563

**Published:** 2025-05-26

**Authors:** Yunchang Li, Xiaojing Zhang, Zhenyu Xia, Yue Hao

**Affiliations:** Institute of Apicultural Research, Chinese Academy of Agricultural Sciences, Beijing 100093, China; yunchangli1993@163.com (Y.L.); 15624955894@163.com (X.Z.); 18706381570@163.com (Z.X.)

**Keywords:** 10-hydroxy-2-decenoic acid, mandibular gland, fatty acid metabolism, acyl-CoA Delta(11) desaturase

## Abstract

10-Hydroxy-2-decenoic acid (10-HDA), a major fatty acid (FA) component of royal jelly, is synthesized in the mandibular glands (MGs) of worker honeybees. Despite its well-documented nutritional and therapeutic significance, the biosynthetic pathway and regulatory mechanisms of 10-HDA production remain largely unresolved. In this study, the molecular basis of 10-HDA biosynthesis and regulation in the MGs of newly emerged bees (NEBs), nurse bees (NBs), and forager bees (FBs) were investigated using RNA sequencing and weighted gene co-expression network analysis (WGCNA). A five-step biosynthetic pathway for 10-HDA was proposed, and cross-species analysis of *Apis mellifera* and *A. cerana* revealed the conserved expression patterns of 15 key enzymes involved. Functional validation via RNA interference (RNAi) demonstrated that knockdown of acyl-CoA Delta(11) desaturase (*d11ds*, LOC551527), a key enzyme in FA desaturation, led to a 50% reduction in 10-HDA levels. Protein–protein interaction (PPI) network analysis further identified transcriptional regulators Kay and Drep-2 as potential modulators of 10-HDA metabolism. This study provides the first comprehensive mechanistic model of 10-HDA biosynthesis in honeybee MGs and highlights the labor-specific regulation of FA metabolism. These findings offer promising genetic targets for improving the royal jelly quality through genetic technology.

## Simple Summary

Honeybee workers utilize specialized mandibular glands (MGs) to produce 10-hydroxy-2-decenoic acid (10-HDA), a fatty acid unique and critical for royal jelly. Despite its nutritional and therapeutic significance, the molecular basis of 10-HDA biosynthesis remained unresolved. This study elucidated the production mechanism by analyzing gene expression patterns in honeybee glands across developmental stages and comparing these processes between two closely related species (*Apis mellifera* and *A*. *cerana*). We identified five interconnected metabolic steps governing 10-HDA synthesis, revealing conserved regulatory patterns in both species. MGs with elevated 10-HDA output exhibited enhanced activity of fatty acid-processing proteins while suppressing a key enzyme (encoded by *d11ds*, LOC551527) responsible for modifying fatty acid structures, reducing the 10-HDA levels by over 50%, directly linking this gene to biosynthesis. PPI network analysis further highlighted transcriptional regulators Kay and Drep-2 as potential modulators of 10-HDA metabolism. These findings establish the first complete model of 10-HDA production, demonstrating how the fatty acid metabolism of honeybee MGs adapts to worker roles.

## 1. Introduction

Honeybees are key pollinators and resource insects. By facilitating pollen transfer, they support plant reproduction and ecosystem diversity while producing honey, royal jelly (RJ), propolis, and beeswax, driving industries in food, pharmaceuticals, and cosmetics. This dual role connects natural ecosystems with human economies [1].

Among these honeybee products, RJ, a unique substance produced by nurse bees in beehives, stands out for its nutritional and bioactive properties, supporting both colony development and offering health benefits to humans [2]. RJ is synthesized and secreted primarily by the hypopharyngeal glands (HGs) and mandibular glands (MGs) of nurse bees. The HGs are mainly responsible for producing major royal jelly proteins (MRJPs), while the MGs predominantly secrete lipid constituents including 10-HDA [3,4]. A hallmark of RJ is its high content of medium- and short-chain unsaturated fatty acids (FAs), predominantly synthesized in the MGs of worker bees [4]. Among these FAs, 10-HDA is essentially exclusive to RJ in nature, especially at the high concentrations observed. Beyond its nutritional role in colonies, 10-HDA demonstrates diverse pharmacological activities including antimicrobial, immunomodulatory, anticancer, and anti-inflammatory effects as well as lifespan extension [5,6]. Consequently, 10-HDA is widely recognized as a marker of RJ quality and commercial value, as formalized in the latest international standard of Royal jelly production [7]. RJ is primarily studied in *Apis mellifera*, with the content of 10-HDA in RJ varying geographically and among different populations, with reported concentrations of 1.53% in Eastern Croatia, 1.8% in Thailand, 2.0% in China, and up to 2.6% in France [8]. Other honeybee species, such as *A. cerana*, *A. florea* and *A. dorsata*, also produce RJ, though the 10-HDA content remains largely uncharacterized [9,10].

The synthesis of 10-HDA by MGs is closely linked to the age-dependent division of labor in honeybees [11,12]. Ultrastructural analyses have revealed that the MG cells of NBs are densely packed with mitochondria, smooth endoplasmic reticulum, and secretory vesicles, supporting active lipid synthesis. In contrast, the MGs of FBs’ shift toward lipid storage [13]. Beyond caste-dependent morphological specialization, 10-HDA synthesis in MGs dynamically correlates with labor transitions. NEBs produce little 10-HDA, whereas NBs (typically 6–12 days old) specialize in RJ secretion and exhibit high 10-HDA production. Although FBs no longer secrete RJ, their MGs continue synthesizing 10-HDA during the early foraging stage, peaking at approximately 25 days of age. However, in the later foraging stage (after 25 days of age), the 10-HDA levels gradually decline [11,14]. These shifts in MG functions are likely influenced by broader lipid metabolism changes across developmental stages. Our previous study demonstrated significant differences in brain lipidomes among NEBs, NBs, and FBs [12], suggesting that systemic lipid regulation may play a role in coordinating both brain function and MG FA synthesis during labor transitions.

The previous study simply described 10-HDA biosynthesis as the following process: the synthesis of stearic acid, the ω-hydroxylation of stearic acid, and incomplete β-oxidation [15,16,17,18]. It has been identified that the biosynthesis of 10-HDA is regulated by a combination of dietary and molecular factors. Nutritional supplementation with extra pollen and precursor FAs such as 10-hydroxydecanoic acid, stearic acid, and decanoic acid have been shown to influence 10-HDA levels [19,20,21]. At the molecular level, several key enzymes are involved, including 3-ketoacyl-CoA thiolase (KAT), cytochrome P450 enzyme CYP6AS8, and electron transfer flavoprotein subunit beta (ETF-β) [11,22,23,24]. KAT plays a central role in the β-oxidation of FAs, and RNAi-mediated knockdown of kat significantly reduces 10-HDA synthesis in honeybees [22]. Similarly, CYP6AS8 is implicated in the hydroxylation of FAs [23,24], and suppression of cyp6as8 via RNAi results in a marked decline in 10-HDA production [23]. ETF-β, which is essential for electron transport during FA metabolism [11], further highlights the enzymatic interdependence required for efficient 10-HDA biosynthesis. Collectively, these findings underscore the synergistic contributions of dietary precursors and key metabolic enzymes in the regulation of 10-HDA production. Additionally, our previous work further identified acyl-CoA binding protein (ACBP) as a key regulator of 10-HDA biosynthesis [25]. Collectively, these studies provide significant insights into the molecular and biochemical mechanisms underlying 10-HDA production in honeybees. However, further investigations are required to elucidate the precise biosynthetic pathways and the gene co-regulatory networks involved in its metabolism.

In this study, we performed transcriptomic profiling of NEBs, NBs, and FBs—stages with distinct 10-HDA levels. By integrating previous findings with our data, we propose a comprehensive biosynthetic framework for 10-HDA and identify the core regulatory genes through cross-species validation (*A. mellifera* and *A. cerana)*. These two species share a common evolutionary ancestor and exhibit similar colony structures and physiological processes including RJ secretion and MG development. These findings offer valuable insights into the genetic basis of 10-HDA production and lay the groundwork for optimizing RJ quality through targeted genetic interventions.

## 2. Materials and Methods

### 2.1. Sample Collection

Samples of *A. mellifera* and *A. cerana* were collected from six colonies (three per species) at the apiary of the Institute of Apicultural Research, Chinese Academy of Agricultural Sciences, Beijing, China. Colonies with similar conditions were used as biological replicates. Frames containing capped brood at approximately day 20 of development were removed and incubated at 34 °C and 70% relative humidity in a climate-controlled chamber (Zhongyiguoke technology Co., Beijing, China).

Honeybees were classified based on their behavioral roles. Frames containing capped brood at the pre-eclosion stage were collected from field colonies (*A. mellifera* and *A. cerana*) and incubated at 34 °C and 70% relative humidity. NEBs were collected within 24 h of emergence. To obtain NBs and FBs, NEBs were marked on the thorax with non-toxic paint and returned to their original hives. Ten-day-old bees exhibiting nursing behavior within brood cells were identified and collected as NBs. The FBs were collected at the hive entrances, identified by the presence of pollen loads on their hind legs [26].

Bees were immobilized on a beeswax dish with dry ice to maintain low temperatures. MGs were dissected under a stereomicroscope and immediately frozen in liquid nitrogen, then stored at −80 °C for further analysis.

### 2.2. Morphology Observation

MG dissections were conducted following the method described by Zhang [12]. Individual honeybee workers (*A. mellifera*) were immobilized on a beeswax dish using dry ice to maintain a low temperature and minimize movement. The head capsule was carefully removed using fine forceps to expose the MGs. Surrounding tissues were gently detached to ensure precise dissection [12]. The mandibles, along with their associated MGs, were then carefully dissected and transferred onto glass slides prior to the separation of the mandibles. The dissected glands were photographed under a stereomicroscope, ZEISS Stemi 508 (Carl Zeiss AG, Oberkochen, Germany) with an attached microscope E3ISPM industrial digital camera (Tiannuoxiang Technology Co., Beijing China). Gland dimensions were measured using ImageView v2.4.0 (BestScope Co., Beijing, China).

For detailed morphological analysis, scanning electron microscopy (SEM) was performed according to the protocol of Hu et al. [14]. The MGs were first fixed in 2.5% glutaraldehyde, then dehydrated through a graded ethanol series. After dehydration, samples underwent critical point drying to ensure complete desiccation. The dried specimens were then coated with a thin layer of gold in a vaporizer and imaged using a Hitachi SU8000 field-emission SEM (Hitachi High-Tech, Tokyo, Japan), where the working distance was 9200 μm and the emission current was 8600 nA.

### 2.3. Extraction of 10-HDA from MGs

The extraction and detection of 10-HDA in the MGs of worker bees (*A. mellifera*) at different ages (NEB, NB, and FB) followed the method described by Mohamed A. K. [27]. MGs dissected from 60 worker bees of the same colony were homogenized in 1 mL chromatographic-grade methanol using a Fluko homogenizer (Fluko Equipment Co. Ltd., Shanghai, China) with five cycles of 8-second homogenization followed by 8-second intervals. The homogenate was centrifuged at 12,000 rpm (4 °C, 15 min), and the supernatant was mixed with 200 μL internal standard solution (methyl hydroxybenzoate, 1280 μg/mL). Methanol was added to adjust the final volume to 2 mL, after which the mixture was sonicated for 15 min. The solution underwent secondary centrifugation at 5000 rpm (4 °C, 15 min), and 1 mL of the clarified extract was filtered through a 0.22 μm membrane using a disposable syringe prior to chromatographic analysis.

### 2.4. Detection of 10-HDA by High-Performance Liquid Chromatography (HPLC)

HPLC analysis was performed using an Agilent 6495 Triple Quadrupole LC/MS system (Agilent Technologies, Santa Clara, CA, USA) equipped with a ZORBAX Eclipse XDB C18 column (5 μm, 4.6 mm × 150 mm). The mobile phase consisted of methanol and 0.05% phosphoric acid (50:50, *v*/*v*) with a flow rate of 0.8 mL/min. The injection volume was 1 μL in positive ion mode and 2 μL in negative ion mode. The maximum absorption peak for 10-HDA was detected at 210 nm. Three biological replicates were conducted for each sample group.

### 2.5. Total RNA Extraction and Transcriptome Analysis

The total RNA of MGs of *A. mellifera* (txid: 7460) and *A. cerana* (txid: 7461) were extracted using TRIzol reagent (Invitrogen, Waltham, MA, USA). The concentration and integrity of the RNA samples were determined using Nanodrop2000 (Thermo Fisher Scientific Inc., Waltham, MA, USA) and a Bioanalyzer 2100 system (Agilent Technologies, Santa Clara, CA, USA) with acceptable purity ratios of A260/A280 between 1.8 and 2.0 as well as an RNA integrity number (RIN) > 8.0.

For each sample, 3 μg of RNA was sent to the sequencing facility (Majorbio Bio-Pharm Technology Co. Ltd., Shanghai, China) for RNA purification, reverse transcription, and library construction, following the NEBNext^®^ Ultra™ RNA Library Prep Kit for Illumina (New England Biolabs, Ipswich, MA, USA). Sequencing analysis was conducted on the Illumina HiSeq X Ten platform. Raw sequencing reads (FASTQ format) were processed using a Perl (v5.36.0) script to remove low-quality reads (Q20 < 50%), reads containing sequencing adapters, and reads with poly-N bases. The cleaned high-quality reads were assessed for base quality (Q30) and GC content. The filtered reads were aligned to the reference genome using TopHat v2.0.12 with default parameters, assembly Amel 4.5 for *A. mellifera,* and ACSNU-2.0 for *A. cerana*. Aligned reads were quantified using HTSeq v0.6.1, and the gene expression levels were normalized TPM (transcripts per million).

Differential gene expression analysis was conducted using DESeq2 v1.46.0. Genes were considered significantly differentially expressed (DEGs) if the adjusted *p-*value < 0.05 and |log_2_ fold change (FC)| ≥ 1.

Principal component analysis (PCA) was conducted to evaluate transcriptional variation in MGs across worker labors (NEBs, NBs, and FBs of *A. mellifera*). Raw read counts were normalized via log_2_(TPM + 1) transformation prior to analysis. Using the prcomp function in R (v4.1.2) with default parameters, PCA was performed on the variance–covariance matrix, with the first two principal components (PC1 and PC2) retained for visualization. Variance contributions were calculated as the ratio of each component’s eigenvalue to total variance. Clustering patterns were visualized using ggplot2 (v3.5.1).

Weighted gene co-expression network analysis (WGCNA) was performed to identify co-expressed gene modules associated with distinct worker labors (NEBs, NBs, and FBs of *A. mellifera*) and to correlate these gene modules with phenotypic variation in 10-HDA levels. Transcriptomic data from NEBs, NBs, and FBs were normalized via log_2_(TPM + 1) transformation, and lowly expressed genes (read counts < 3 in ≥2 replicates) were filtered. Using the WGCNA R package (v1.73), a signed adjacency matrix was constructed from the Pearson correlation coefficients between the gene expression profiles. A soft-thresholding power (β = 7) was selected to achieve the scale-free topology (R^2^ > 0.4). The adjacency matrix was converted to a topological overlap matrix (TOM), and gene modules were identified through hierarchical clustering with dynamic tree cutting. Module-trait correlations were calculated by correlating the module eigengenes with the 10-HDA levels. Functional enrichment of key modules was analyzed using the DAVID database, with Kyoto Encyclopedia of Genes and Genomes (KEGG) pathways considered significant at *p* < 0.05. Gene Ontology Biological Process (GOBP) [28] and KEGG pathway enrichment analyses [29] were conducted using DAVID v6.8 to identify biological processes associated with MG function across different labors (NEBs, NBs, and FBs of *A. mellifera*). Significantly enriched terms were determined with a Benjamini–Hochberg-adjusted *p* < 0.05. Protein–protein interaction (PPI) networks of DEGs were constructed using STRING v12.0, incorporating interaction evidence from text mining, experiments, databases, neighborhood, and gene fusion. A medium confidence threshold (interaction score ≥ 0.15) was applied to filter network edges.

### 2.6. RNA Interference (RNAi)

RNA interference (RNAi) was performed following the method described by Yong Z [30]. The *d11ds*-specific dsRNA (Forward: TAATACGACTCACTATAGGACATAGGCTGGTTAATGGTCCGA; Reverse: TAATACGACTCACTATAGGTCGATCCGTCTCCAGTTCTAGCT) and GFP-specific dsRNA (Forward: TAATACGACTCACTATAGGAGAGGGTGAAGGTGATGCAA; Reverse: TAATACGACTCACTATAGGTGGTCTGCTAGTTGAACGCT) sequences were designed using siDirect (http://sidirect2.rnai.jp/, accessed on 11 January 2025) and DSIR (http://biodev.extra.cea.fr/DSIR/DSIR.html, accessed on 11 January 2025). The double-stranded RNAs (dsRNAs) were synthesized in vitro using the T7 RNAi Transcription Kit (Vazyme TR102, Nanjing, China) according to the manufacturer’s instructions. GFP-specific dsRNA was used as a negative control.

Frames with sealed brood from three healthy honeybee colonies (*A. mellifera*) were collected and placed in separate cages inside an incubator at 34 °C and 70% relative humidity. NEBs were collected within 24 h and transferred to cup cages (plastic cups with multiple vent holes) with 50 worker bees per cup. Bees were fed with 50% (*w*/*v*) sucrose solution via a 2 mL syringe (without needles), and artificial beebread (a mixture of water and Brassica napus pollen (Longkou City Fengyuan Honeybee supplies Co., Q/0681LMF004-2020, Longkou, China) was provided at the bottom of the cup. The beebread was changed every 3 days to prevent mildew formation [31].

For dsRNA injection, 6-day-old NBs were anesthetized at 4 °C and injected in the neck region using a Nanoliter 2010 microinjector (World Precision Instruments, Sarasota, FL, USA). Experimental bees received 1 μL of dsRNA solution (3 μg/μL dsRNA- d11ds or dsRNA-GFP) diluted in PBS. Control bees were handled identically but were not injected. After injection, bees were returned to their original cup cages and maintained on 50% (*w*/*v*) sucrose solution for three days. Dead bees were removed daily. The MGs were collected 72 h post-injection for the analysis. Eighteen bees from each treatment group were collected for 10-HDA quantification using HPLC. Nine bees from each treatment group were collected for RNA extraction, and the mRNA expression levels were quantified via quantitative real-time PCR (qRT-PCR).

### 2.7. qRT-PCR Analysis

Total RNA was extracted from the MG samples of NEBs, NBs, and FBs (*A. mellifera*) using TRIzol reagent (Invitrogen, Waltham, MA, USA) following the manufacturer’s instructions. RNA quality and quantity were assessed using a NanoDrop 2000 spectrophotometer (Thermo Fisher, Waltham, MA, USA) and 1.2% agarose gel electrophoresis. First-strand cDNA was synthesized from 1 μg of total RNA using the NovoScript^®^ 1st Strand cDNA Synthesis Kit (Novoprotein E041, Suzhou, China). Quantitative reverse transcription PCR (qRT-PCR) was performed in a 20 μL reaction system using NovoStart^®^ SYBR Green Color qPCR SuperMix (Novoprotein E168, Suzhou, China) with three technical replicates per sample. Reactions were conducted on a LineGene 9600 Plus (Bioer, Hangzhou, China) using the following thermal cycling conditions: 95 °C for 30 s (initial denaturation), 40 cycles of 95 °C for 5 s, and 58 °C for 30 s. Relative gene expression levels were calculated using the 2^−ΔΔCt^ method [32]. *rp49* (Gene ID: 406099), which encodes ribosomal protein L32, was selected as the endogenous control for qRT-PCR analysis. The negative control group (GFP-dsRNA injected bees) was designated as the calibrator, with its mean ΔCt value serving as the baseline for comparisons with the experimental groups. Primer sequences are listed in Table S1.

### 2.8. Statistical Analysis

Results are presented as the mean ± standard error of the mean (SEM), based on at least three independent experiments. Differences between sample groups were evaluated using an unpaired two-tailed Student’s *t*-test, with significance levels set at *p* < 0.05 after applying the Holm–Sidak correction for multiple comparisons. Statistical significance was denoted as follows: * *p* < 0.05, ** *p* < 0.01, *** *p* < 0.001.

## 3. Results

### 3.1. Morphological Differences and 10-HDA Content in MGs of NEBs, NBs, and FBs

**Figure 1:**
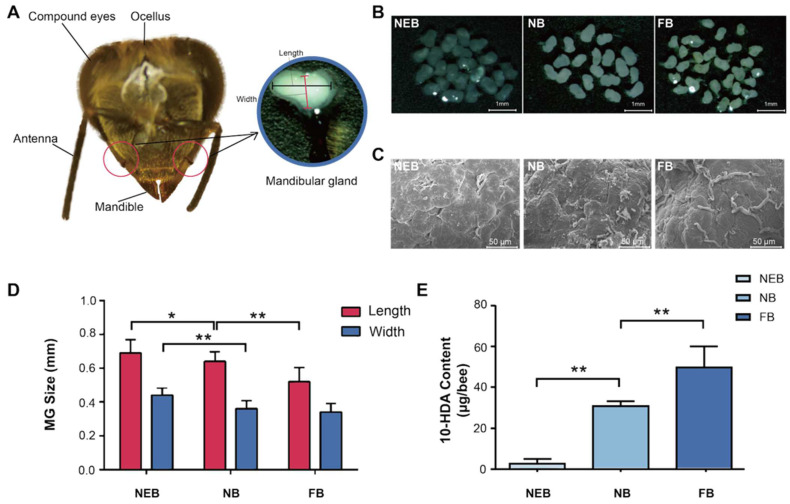
Morphology and secretion were different between the MGs of newly emerged bees (NEBs), nurse bees (NBs), and forager bees (FBs) of *A. mellifera*. (**A**) Head structure of worker bee. (**B**) Light microscopy images of MGs in NEBs, NBs, and FBs (scale bar = 1 mm). (**C**) Scanning electron microscopy (SEM) images showing the surface morphology of MGs in NEBs, NBs, and FBs. (scale bar = 50 μm). (**D**) MG size comparison among NEBs, NBs, and FBs. (**E**) 10-HDA content based on HPLC in the MGs of NEBs, NBs, and FBs, error bars represent data from bees across three colonies with three biological replicates. Statistical significance was determined by the two-tailed unpaired Student’s *t*-test. ** and * indicate statistical significance at *p* < 0.01 and *p* < 0.05, respectively.

The MGs of honeybees are located on both sides of the head, adjacent to the base of the mandibles, and have a sac-like structure (Figure 1A). Gross anatomical analysis revealed distinct age-dependent morphological variations in the MGs of *Apis mellifera*: NEBs exhibited flattened, translucent MGs with minimal secretory activity; NBs demonstrated fully developed MGs containing abundant milky secretion, indicating peak glandular development and secretory function; and FBs showed significantly reduced MG size and secretory content, consistent with physiological transition to foraging behavior (Figure 1B). Scanning electron microscopy (SEM) results showed that the MGs of NEBs, NBs, and FBs exhibited a higher abundance of duct structures on their surfaces. This structural feature is consistent with the changes in the secretory function associated with age and behavioral differentiation among worker bees (Figure 1C).

Morphometric analysis revealed a significant age-related, caste-dependent reduction in MG size of *A. mellifera*, with dimensions (length/width) of 0.69 ± 0.02/0.44 ± 0.01 mm in NEBs, 0.64 ± 0.03/0.36 ± 0.02 mm in NBs, and 0.52 ± 0.01/0.34 ± 0.01 mm in FBs (Figure 1D, Table S2). Correspondingly, the 10-HDA content increased markedly with age: 3.07 ± 1.93 μg/bee in NEBs, 31.17 ± 2.02 μg/bee in NBs (10.2-fold), and 50.24 ± 9.90 μg/bee in FBs (16.4-fold) (Figure 1E, Table S3). These results establish a strong correlation between MG morphology, 10-HDA production, and age-related castes in worker bees.

### 3.2. Transcriptomic Profiling of MGs from NEBs, NBs and FBs of A. mellifera

**Figure 2:**
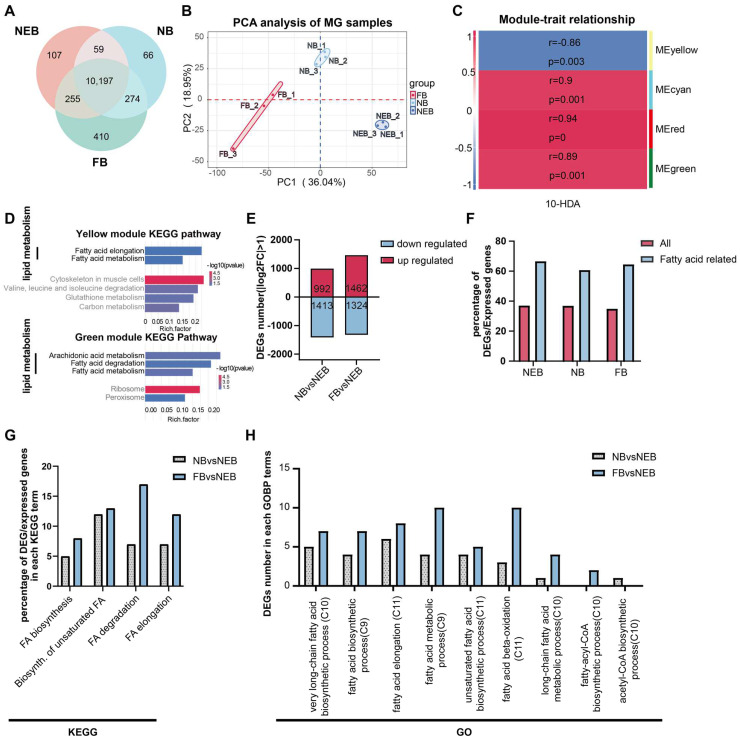
Functional annotation of differentially expressed genes (DEGs) in the MGs of NEBs, NBs, and FBs in *Apis mellifera*. (**A**) Distribution of expressed genes in the MGs of NEBs, NBs, and FBs (genes with read counts ≥ 3 in at least two out of three samples are considered expressed). (**B**) PCA analysis of the MG of the NEB, NB, and FB samples. (**C**) Correlation between the four gene modules and 10-HDA content (r indicates the correlation coefficient, *p* indicates the result of the Pearson’s correlation test) based on WGCNA analysis. (**D**) KEGG enrichment of the genes from yellow and green modules. (**E**) Number of up- and downregulated DEGs in various comparisons (NBs vs. NEBs, FBs vs. NEBs). (**F**) Proportion of DEGs involved in fatty acid metabolism in the MGs of NEBs, NBs, and FBs. (**G**) Proportion of DEGs associated with fatty acid metabolism in each KEGG term. (**H**) Proportion of DEGs associated with fatty acid metabolism in each GO term.

A total number of 111,368 genes were identified with criteria of the gene read counts ≥ 3 in at least two biological replicates. Among these, 10,197 genes were constitutively expressed across all castes (Figure 2A, Table S4). Principal component analysis (PCA) revealed a distinct separation between the NEBs, NBs, and FBs (Figure 2B), confirming robust biological reproducibility and labor-dependent transcriptional divergence.

The WGCNA of MG expression was performed (Figure S1A,B, Table S5) to resolve the 10-HDA phenotype-specific modules as shown in Figure 2C; four key modules (yellow, cyan, red, green) showed significant correlations (|r| > 0.85, *p* < 0.05) with the 10-HDA levels in NEBs, NBs, and FBs (Table S6).

To investigate the functional relevance of genes in the four modules, we performed KEGG enrichment analysis on each module’s gene set. Lipid metabolism pathways were prominently enriched in the yellow and green modules (Figure 2D, Table S7). The yellow module’s gene set showed significant enrichment in six KEGG pathways (*p* < 0.05), including two Lipid metabolism-related pathways: Fatty acid elongation and Fatty acid metabolism. Similarly, the green module’s gene set was enriched in five KEGG pathways, three of which were associated with lipid metabolism: arachidonic acid metabolism, fatty acid degradation, and fatty acid metabolism. These findings indicate that lipid metabolism predominantly underlies the functional relevance of the yellow and green modules to 10-HDA content. Enrichment results for the red and cyan modules are presented in Supplementary Figure S1C,D.

Comparative analysis identified 2405 DEGs in the comparison of NBs versus NEBs (992 genes upregulated in NBs), and 2786 DEGs in FBs versus NEBs (1462 genes upregulated in FBs) (Figure 2E, Table S8). To determine whether DEGs in the MGs of worker bees with distinct labor roles were enriched in FA-related pathways, we analyzed the FA-associated genes curated from the KEGG database and GOBP terms in *A. mellifera* (Table S9). Given that lipid metabolism, particularly FA metabolism, is linked to 10-HDA content (Figure 2D) and 10-HDA itself is a FA, we focused on FA-related pathways. Consistent with established roles of MG in FA synthesis [11], the FA-related genes showed disproportionately high differential expression. While an average 36.8% of all expressed genes were DEGs (*p* < 0.05; Figure 2F, red bars, Table S9), a striking 62.3% of the FA-related genes were differentially expressed (*p* < 0.05, Figure 2F, blue bars, Table S9). To investigate the functional implications of DEGs identified in the NBs vs. NEBs and FBs vs. NEBs comparisons within FA metabolism, we analyzed their distribution across relevant KEGG pathways and GOBP terms. KEGG pathway analysis revealed a significant enrichment of DEGs in two critical pathways: 25 DEGs were mapped to the biosynthesis of unsaturated fatty acids (12 from NBs vs. NEBs, 13 from FBs vs. NEBs) while 24 DEGs were associated with fatty acid degradation (7 from NBs vs. NEBs, 17 from FBs vs. NEBs) (Figure 2G, DEGs of NBs vs. NEBs are in the grey dot bar, DEGs of FBs vs. NEBs are in the blue bar, Table S10). Notably, the FB vs. NEB DEGs showed a predominant distribution in fatty acid degradation compared with their NB vs. NEB counterparts. GOBP term analysis demonstrated differential distribution patterns of DEGs in key biological processes: 14 DEGs were mapped in fatty acid elongation processes (6 from NBs vs. NEBs in the grey dot bar, 8 from FBs vs. NEBs in the blue bar), another 14 DEGs participated in general fatty acid metabolism (4 from NBs vs. NEBs, 10 from FBs vs. NEBs). The fatty acid β-oxidation process contained 13 DEGs, displaying a marked contrast between the comparison groups (3 from NBs vs. NEBs vs. 10 from FBs vs. NEBs) (Figure 2H, DEGs of NBs vs. NEBs are in the grey dot bar, DEGs of FBs vs. NEBs are in the blue bar, Table S11). These distribution patterns suggest distinct regulatory roles of the two comparison groups in specific fatty acid metabolic processes.

### 3.3. 10-HDA Biosynthesis Pathway in MG

**Figure 3:**
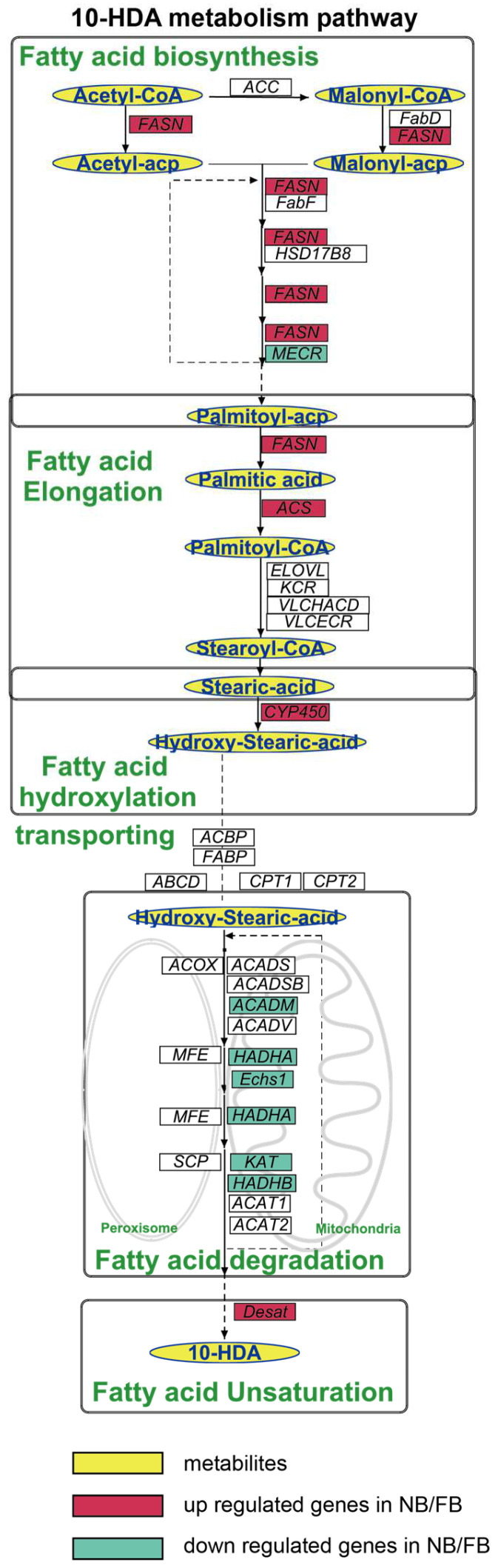
Diagram of the 10-HDA metabolism pathway, highlighting the key intermediates and final products in the blue oval with a yellow background. Genes are indicated in italics, with DEGs in both *A. mellifera* and *A. cerana* shown with a colored background; the red background indicates the upregulated gene in NBs/FBs relative to NEBs; the green background indicates the downregulated gene in NBs/FBs relative to NEBs. *ACC, acetyl-CoA carboxylase; FASN, fatty acid synthesis; FabD: malonyl-CoA-acyl carrier protein transacylase, mitochondrial; FabF: 3-oxoacyl-[acyl-carrier-protein] synthase, mitochondrial; HSD17B: estradiol 17-beta-dehydrogenase 8; MECR: enoyl-[acyl-carrier-protein] reductase, mitochondrial; ACSL: long-chain acyl-CoA ligase; ELOVL: elongation of very long chain fatty acids protein; VLCHACD: very-long-chain (3R)-3-hydroxyacyl-CoA dehydratase; VLCECR: very-long-chain enoyl-CoA reductase; CYP450: cytochromeP450; ACOX, acyl-CoA oxidases; MFE, peroxisomal multifunctional enzyme; SCP, non-specific lipid-transfer protein; ABCD: ATP-binding cassette sub-family D; CPT 1: carnitine O-palmitoyltransferase 1; CPT 2: carnitine O-palmitoyltransferase 2; ACAD, acyl-CoA dehydrogenases; HADHA, α-subunit of the trifunctional enzyme; Echs, enoyl coenzyme A hydratase; KAT, 3-ketoacyl-CoA thiolase; HADHB, trifunctional enzyme subunit beta.*

To elucidate the mechanism of 10-HDA production, we proposed a revised 10-HDA biosynthetic pathway, based on the integration of the results of previous isotopic tracer studies [15,16,17], KEGG and GOBP annotations, and five coordinated processes operated sequentially (Figure 3). The pathway initiates with de novo FA biosynthesis, driven by acetyl-CoA carboxylase (ACC) and fatty acid synthase (FASN), which catalyze sequential two-carbon additions to synthesize palmitoyl-ACP. Subsequently, palmitic acid undergoes elongation to stearoyl-CoA through a multi-enzyme cascade involving acyl-CoA synthetases, elongases, and reductases. The elongated stearic acid is then ω-hydroxylated via CYP450-mediated catalysis to form ω-hydroxystearic acid. Subcellular transport and β-oxidation follow, with mitochondrial and peroxisomal transporters facilitating FA shuttling, while oxidases and dehydrogenases degrade acyl-CoA intermediates through compartment-specific β-oxidation cycles. Finally, acyl-CoA Δ11-desaturase introduces double bonds into saturated chains, completing the pathway to generate 10-HDA.

The pathway comprised 136 genes, among which 46/69 genes were identified as DEGs between NBs vs. NEBs/FBs vs. NEBs of *A. mellifera* according to our transcriptome analysis (FC > 2 or FC < 0.5, *p*adj < 0.05). Key enzymes and regulatory proteins associated with each step are systematically summarized in Table 1.

**Figure 4:**
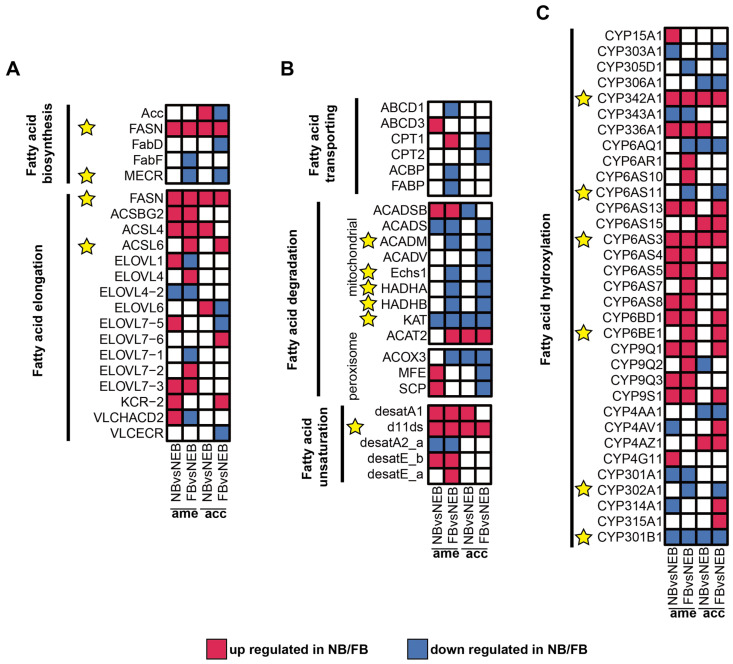
Expression of DEGs related to 10-HDA metabolism in the MGs of *A. mellifera* and *A. cerana*. (**A**–**C**) Expression levels of DEGs in 6 modules related to 10-HDA metabolism between NBs/FBs and NEBs: fatty acid biosynthesis, fatty acid elongation (**A**); fatty acid transporting, fatty acid degradation, fatty acid unsaturation (**B**); fatty acid hydroxylation (**C**). Upregulated genes in NBs/FBs relative to NEBs are shown in red, downregulated genes in NBs/FBs relative to NEBs are shown in blue, and non-significantly different genes are shown in white; the yellow star sign indicates the conserved DEGs between *A. mellifera* and *A. cerana*.

To identify core regulatory genes in 10-HDA biosynthesis, conserved regulatory patterns between *A. mellifera* and *A. cerana*, consistent with their shared RJ secretion biology [33,34], were obtained from a comparative analysis of DEGs (|log_2_FC| ≥ 1, *p*adj < 0.05) between NBs and NEBs, and the DEGs between FBs and NEBs; the transcriptome profile of *A. cerana* is available in the Supplementary Materials and Figure S2. As shown in Figure 4A–C, red tiles show the upregulation of DEGs in NB/FB; blue tiles indicate the downregulation of DEGs in NB/FB, and those with non-significance are labeled with white tiles, the yellow star sign indicates the conserved DEGs between *A. mellifera* and *A. cerana* (see Table S12, Figure S2, and Supplementary Materials for more details). Genes showing parallel expression trends in both species were prioritized in Table 2 and yellow stars in Figure 4: in FA biosynthesis, FASN showed consistent upregulation in both nurse bees (NBs) and forager bees (FBs) of the two species, whereas enoyl-[acyl-carrier-protein] reductase, specifically mitochondrial (MECR), was downregulated specifically in FBs. For fatty acid elongation, long-chain acyl-CoA ligase 6 (ACSL6) exhibited elevated expression levels in FBs across both species. Mitochondrial β-oxidation-related genes (acyl-CoA dehydrogenases, mitochondrion (ACADM), enoyl coenzyme A hydratase 1 (ECHS1), α-subunit of the trifunctional enzyme (HADHA), and β-subunit of the trifunctional enzyme (HADHB)) displayed coordinated downregulation in FBs, consistent with the observed suppression of KAT in both NBs and FBs—a pattern aligning with previous findings on the KAT-mediated regulation of 10-HDA biosynthesis [22]. Notably, the Δ11-desaturase homolog (*d11ds*) was significantly upregulated in NBs/FBs of both species, implicating its potential role in FA unsaturation. ω-hydroxylation-associated cytochrome P450 genes exhibited divergent regulation: CYP342A1 and CYP6AS3 were upregulated in NBs/FBs, while CYP6AS11 and CYP302A1 showed FB-specific downregulation. In contrast, CYP301B1 was suppressed in both NBs and FBs, whereas CYP6BE1 demonstrated FB-specific upregulation.

The *d11ds* was prioritized for further investigation based on two key findings from the cross-species comparative analysis (Figure 4B, Table 1). First, *d11ds* exhibited conserved upregulation in both NBs and FBs of *A. mellifera* and *A. cerana*, suggesting a phylogenetically conserved role in FA metabolism. Second, its coordinated expression pattern across species implicated *d11ds* in addressing unresolved enzymatic steps related to FA unsaturation during 10-HDA biosynthesis—a critical functional component of RJ. This dual evidence of evolutionary conservation and pathway-specific relevance positioned *d11ds* as a strong candidate for mechanistic validation.

### 3.4. Functional Validation of d11ds in 10-HDA Biosynthesis

**Figure 5:**
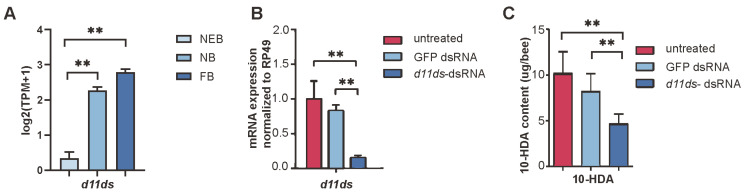
Knockdown of *d11ds* reduces 10-HDA production in the MGs of *A. mellifera* worker bees. (**A**) Expression of *d11ds* in the MGs of worker bees. (**B**) Expression levels of *d11ds* and other desaturases in RNAi samples. (**C**) Decreased 10-HDA production in the *d11ds* RNAi samples. The mean of three biological replicates is plotted with error bars representing SEM. Statistical significance was determined by the two-tailed unpaired Student’s *t*-test. ** indicates statistical significance at *p* < 0.01.

In *A. mellifera*, *d11ds* exhibited developmentally upregulated expression in worker MGs, with the transcript levels significantly increasing across caste transitions (Figure 5A, see Table S5). To validate its role in 10-HDA metabolism, we performed RNAi-mediated knockdown targeting *d11ds*. Compared with the untreated controls and GFP dsRNA-injected groups, the *d11ds* transcript levels decreased by 68.2%; see Table S13 (*p* < 0.01, *t*-test), in the dsRNA-treated MGs (Figure 5B). HPLC quantification revealed corresponding reductions in 10-HDA content: knockdown samples retained 45.6% of the untreated control levels and 56.5% of the GFP dsRNA-injected levels; see Table S14 (Figure 5C). These results confirm *d11ds* as a critical regulator of 10-HDA biosynthesis in worker bee MGs.

### 3.5. Transcriptional Regulation of 10-HDA Biosynthesis

**Figure 6:**
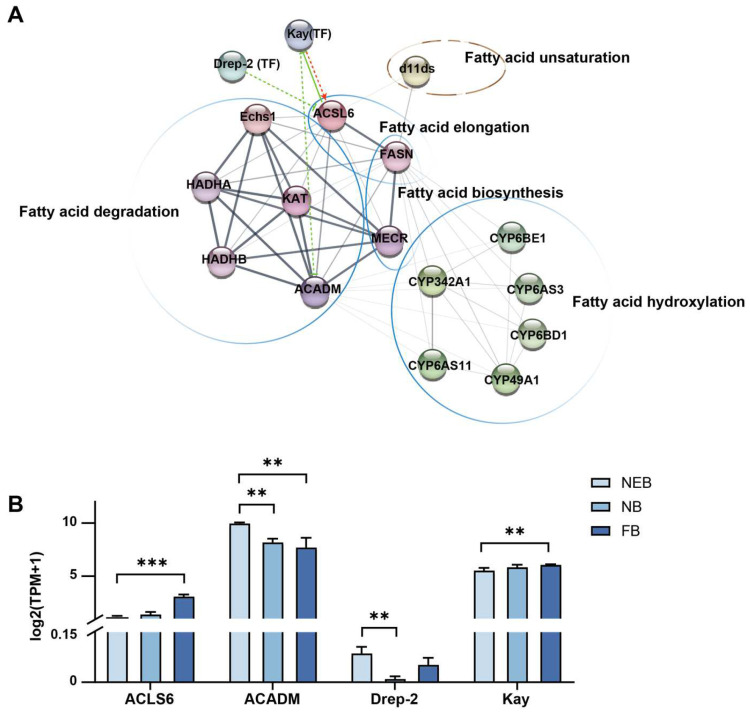
The PPI network of the 10-HDA related genes and the differently expressed transcription factors between NEBs vs. NBs and NEBs vs. FBs in *A. mellifera*. (**A**) PPI network of the 10-HDA related genes and the differently expressed transcription factors between NEBs, NBs, and FBs (see Table 2) was constructed using STRING tools. Transcription factors (TFs) are represented by “(TF)”, while the remaining proteins are indicated by spherical nodes. Line thickness (gray scale) represents the interaction strength. Regulatory relationships are categorized as follows: red lines denote activation, green solid lines indicate inhibition, and dashed lines suggest potential regulatory associations. Enrichment results of KEGG are indicated by circles. (**B**) The expression level of Kay and Drep-2 coding genes and their target genes related to 10-HDA biosynthesis identified by RNA-Seq. The mean of three biological replicates was plotted with error bars representing SEM. Statistical significance was determined by the two-tailed unpaired Student’s *t*-test. ** and *** indicate statistical significance at *p* < 0.01 and *p* < 0.001, respectively.

To identify transcriptional regulators of 10-HDA production, we analyzed 81 transcription factors (TFs) expressed in MGs in *A. mellifera* across developmental stages. Differential expression analysis revealed the following: 8 TFs were differentially expressed (DE) in NEBs vs. NBs (*p*adj < 0.05) and 19 TFs were DE in NEBs vs. FBs (*p*adj < 0.05) (see Table S15). PPI networks integrating DE TFs and core 10-HDA biosynthesis genes (Table 1) were constructed using STRING (v12.0; interaction score ≥ 0.15) and visualized via Cytoscape (v3.10.3) with StringApp (v2.2); see Table S16 for the interaction scores in detail (Figure 6A). In the interaction network diagram, the interaction was based on the string database, originating from the experimentally determined, gene fusion, and literature reported. Network topology analysis identified two hub TFs: Kay, positively correlated with ACSL6 (interaction score = 0.165) and negatively correlated with ACADM (interaction score = 0.19); and Drep-2, negatively regulated ACSL6 (interaction score = 0.201). Kay is a homolog of the human proto-oncogene and subunit of AP-1 in *Drosophila*, critical for embryonic development [35,36], Notably, human ACSL6 homologs have been reported to inhibit the activity of Kay in humans [37]. Drep-2 is a synaptic protein linked to learning behavior [38]. The expression patterns of these TFs mirrored their regulatory roles (Figure 6B, Table S5): Kay was upregulated in FBs vs. NEBs (FC = 3.13), coinciding with ACSL6 induction (FC = 2.9) and ACADM suppression (FC = 0.22). Drep-2 was downregulated in FBs vs. NEBs (FC = 0.25) and inversely correlated with ACSL6 expression. These results implicate Kay and Drep-2 as key transcriptional regulators coordinating FA metabolism during 10-HDA biosynthesis.

## 4. Discussion

This study systematically elucidated the molecular mechanisms underlying 10-HDA biosynthesis in honeybee MGs. By integrating comparative transcriptomics, functional validation, and pathway modeling, we provided mechanistic insights into the synthesis of this economically pivotal compound.

Prior to 1995, 10-HDA was conventionally classified as a typical metabolic intermediate in FA metabolism. This paradigm shifted when isotopic labeling experiments by Plettner demonstrated that 10-HDA biosynthesis originates from an 18-carbon fatty acid precursor, thereby redefining its metabolic pathway and biological significance. Subsequent studies revealed that the 18-carbon FA precursor is synthesized de novo from acetate in the MGs of worker bees. These findings established a three-step bifurcated pathway for 10-HDA biosynthesis: (1) de novo synthesis of stearic acid, (2) ω-hydroxylation of the 18-carbon FA, and (3) incomplete β-oxidation to yield the final 10-carbon product. The molecular basis of this pathway was further elucidated through the identification of key regulatory genes. Notably, CYP6AS8 (responsible for 18-carbon hydroxylation) and ketoacyl-CoA thiolase (KAT, involved in β-oxidation) were shown to positively regulate 10-HDA production [18,22,23]. However, existing models fail to account for the unsaturated nature of 10-HDA, as none of these studies addressed the potential role of desaturase enzymes. In this study, we proposed an enhanced biosynthetic model that integrates two critical missing components: (i) FA transport mechanisms between distinct anatomical compartments [25], and (ii) enzymatic desaturation processes required for 10-HDA unsaturation. This refined framework provides comprehensive insights into the spatial coordination and biochemical transformations underlying 10-HDA metabolism.

A critical advancement lies in identifying *d11ds* as the Δ11-desaturase responsible for introducing double bonds during 10-HDA biosynthesis. Acyl-CoA desaturases (Desats), enzymes responsible for introducing double bonds into saturated FAs to generate monounsaturated derivatives, play conserved roles in pheromone biosynthesis across insects [39,40,41]. For instance, *Drosophila melanogaster* Desat1 and Desat2 catalyze the Δ9 desaturation of C16:0 to C16:1 and C14:0 to C14:1, respectively [39], while knockdown of Nlug-desatA2 in *Nilaparvata lugens* reduces the C18:1/C18:0 ratio [41]. Notably, despite the predominance of monounsaturated FAs in *A. mellifera* MG secretions, the functional involvement of Desats in this species remains poorly characterized. Phylogenetic analyses have revealed that all *A. mellifera* Desats belong to the ancestral Δ9 desaturase subfamily [42]. Intriguingly, while most of the desaturases are likely to mediate Δ9 desaturation, some of the desaturases, like desatD, exhibits catalytic specificity for Δ4 bond formation [43], suggesting functional divergence within this conserved enzyme family. Our RNAi experiments conclusively linked *d11ds* to 10-HDA production: a 54% reduction in 10-HDA levels was found in MGs samples of NBs (Figure 5C). This bridges the gap between saturated precursor utilization and final unsaturated 10-HDA, completing the biosynthetic blueprint. The conserved upregulation of *d11ds* in both species (log_2_FC ≥ 3) underscores its evolutionary importance in RJ secretion.

Co-expression network analysis revealed two TFs, Kay and Drep-2, as potential modulators of 10-HDA biosynthesis (Figure 6). Kay is a highly conserved TF whose developmental and proliferative functions are maintained across species from flies to humans. In *Drosophila*, the homolog of Kay is a subunit of AP-1, responsible for embryonic development. In humans, the homolog of Kay is a proto-oncogene *fos*, responsible for cell proliferation and differentiation [35,36]. Its upregulation in high-secreting MGs (log_2_FC = 3.1 in FBs) suggests a role in FA metabolism. Drep-2 is a synaptic protein linked to learning behavior [38], variably expressed during labor transitions, implying labor-specific regulatory adaptation. Our finding suggest that the cell proliferation and development related TF Kay and the behavior regulatory related TF Drep-2 may also play roles in lipid metabolic regulation.

Elucidating 10-HDA biosynthesis in honeybees provides critical insights into colony organization and nutrition for a bee hive. Our findings highlight the molecular mechanisms underlying this process, showing how worker bees upregulate specific metabolic pathways to meet the demands of the larvae and queen bees. This enhances our understanding of honeybee physiology; particularly how genetic factors enable a subset of workers to function as specialized “royal jelly factories”. Based on the reinforced 10-HDA biosynthesis pathway, the upregulation of 10-HDA in RJ might be achieved by the supplementation of intermediate metabolites along the 10-HDA biosynthesis pathway such as palmitic acid and hydroxy stearic acid.

## 5. Conclusions

This study provides a comprehensive elucidation of the molecular mechanisms underlying 10-HDA biosynthesis in honeybee MGs, identifying a five-step biosynthetic pathway and 15 conserved key enzymes across *A. mellifera* and *A. cerana*. Functional validation through RNAi demonstrated the critical role of *d11ds* in 10-HDA production, while PPI network analysis highlighted Kay and Drep-2 as potential transcriptional regulators. By integrating comparative transcriptomics, functional validation, and pathway modeling, our findings offer novel mechanistic insights into the regulation of this economically important compound. These discoveries not only advance our understanding of fatty acid metabolism in honeybees, but also provide promising genetic targets for improving the royal jelly quality through biotechnological interventions.

## Figures and Tables

**Table 1:** Genes associated with 10-HDA biosynthesis.

Process	Entrez Gene ID	Gene Symbol	Function
Fatty acid biosynthesis	552286	*acc*	Acetyl-CoA carboxylase, transcript variant X1
	411959	*fasn*	Fatty acid synthase-like
	412815	*fasn*	Fatty acid synthase
	412286	*f* *abd*	Probable malonyl-CoA-acyl carrier protein transacylase, mitochondrial
	413655	*f* *abf*	3-Oxoacyl-[acyl-carrier-protein] synthase, mitochondrial
	552493	*hsd17b8*	Estradiol 17-beta-dehydrogenase 8
	411662	*mecr*	Probable trans-2-enoyl-CoA reductase, mitochondrial
Fatty acid elongation	411959	*fasn*	Fatty acid synthase-like
	412815	*fasn*	Fatty acid synthase
	551837	*acsbg2*	Long-chain-fatty-acid--CoA ligase ACSBG2, transcript variant X1
	409515	*acsl4*	Long-chain-fatty-acid--CoA ligase 4, transcript variant X1
	412541	*acsl6*	Long-chain-fatty-acid--CoA ligase 6, transcript variant X2
	100578829	*elovl1*	Elongation of very long chain fatty acids protein 1-like
	411692	*elovl4*	Elongation of very long chain fatty acids protein 4-like, transcript variant X2
	552205	*elovl4-2*	Elongation of very long chain fatty acids protein AAEL008004
	725842	*elovl6-like-1*	Elongation of very long chain fatty acids protein 6-like
	113219003	*elovl6-like-2*	Elongation of very long chain fatty acids protein 6-like
	113219005	*elovl6-like-3*	Elongation of very long chain fatty acids protein 6-like
	725031	*elovl6*	Elongation of very long chain fatty acids protein 6
	724552	*elovl7-5*	Elongation of very long chain fatty acids protein AAEL008004-like
	724867	*elovl7-6*	Elongation of very long chain fatty acids protein AAEL008004-like
	409638	*elovl7-1*	Elongation of very long chain fatty acids protein AAEL008004-like, transcript variant X5
	551938	*elovl7-4*	Elongation of very long chain fatty acids protein AAEL008004, transcript variant X4
	413789	*elovl7-2*	Elongation of very long chain fatty acids protein AAEL008004-like
	550828	*elovl7-3*	Elongation of very long chain fatty acids protein AAEL008004-like
	725258	*kcr-2*	Very-long-chain 3-oxoacyl-CoA reductase-like, transcript variant X2
	413078	*vlchacd*	Very-long-chain (3R)-3-hydroxyacyl-CoA dehydratase, transcript variant X2
	100577192	*vlchacd2*	Very-long-chain (3R)-3-hydroxyacyl-CoA dehydratase 2
	725146	*vlcecr*	Very-long-chain enoyl-CoA reductase
Fatty acid hydroxylation	551179	*cyp15a1*	Methyl farnesoate epoxidase
	410405	*cyp18a1*	Cytochrome P450 18a1
	727290	*cyp303a1*	Probable cytochrome P450 303a1
	551223	*cyp305d1*	Probable cytochrome P450 305a1
	408398	*cyp306a1*	Cytochrome P450 306a1, transcript variant X1
	410495	*cyp307b1*	Cytochrome P450 307a1
	724175	*cyp342a1*	Probable cytochrome P450 304a1
	551632	*cyp343a1*	Methyl farnesoate epoxidase, transcript variant X2
	724211	*cyp336a1*	Cytochrome P450 9e2
	408383	*cyp6aq1*	Cytochrome P450 6AQ1, transcript variant X3
	550965	*cyp6ar1*	Probable cytochrome P450 6a14
	413306	*cyp6as1*	cytochrome P450 6AS1
	725159	*cyp6as10*	probable cytochrome P450 6a14
	724946	*cyp6as11*	cytochrome P450 6a2
	413908	*cyp6as12*	cytochrome P450 6A1, transcript variant X1
	551028	*cyp6as13*	Probable cytochrome P450 6a13
	413405	*cyp6as14*	Probable cytochrome P450 6a14
	551197	*cyp6as15*	Probable cytochrome P450 6a15
	725087	*cyp6as16p*	Probable cytochrome P450 6a16
	551626	*cyp6as17*	Probable cytochrome P450 6a17
	411615	*cyp6as2*	Uncharacterized LOC411615
	726690	*cyp6as3*	Uncharacterized LOC726690
	412209	*cyp6as4*	Probable cytochrome P450 6a17
	409677	*cyp6as5*	Cytochrome P450 6AS5
	412936	*cyp6as7*	Cytochrome P450 6A1, transcript variant X2
	413083	*cyp6as8*	Probable cytochrome P450 6a14, transcript variant X2
	107965400	*cyp6as9p*	Uncharacterized LOC107965400
	726646	*cyp6bc1*	Probable cytochrome P450 6a13
	551560	*cyp6bd1*	Cytochrome P450 6k1
	552418	*cyp6be1*	Cytochrome P450 6k1, transcript variant X2
	102656882	*cyp9p1*	Cytochrome P450 9e2-like
	551846	*cyp9p2*	Membralin-like, transcript variant X4
	410492	*cyp9q1*	Cytochrome P450 9e2
	408452	*cyp9q2*	Cytochrome P450 9e2
	408453	*cyp9q3*	Cytochrome P450 9e2
	410490	*cyp9r1*	Uncharacterized LOC410490
	725621	*cyp9s1*	Uncharacterized LOC725621
	100577883	*cyp4aa1*	Cytochrome P450 4aa1-like
	552679	*cyp4av1*	Cytochrome P450 4c3
	413833	*cyp4az1*	Cytochrome P450 4C1
	409469	*cyp4g11*	Cytochrome P450 4G11
	413730	*cyp301a1*	Probable cytochrome P450 301a1, mitochondrial
	727118	*cyp302a1*	Cytochrome P450 302a1, mitochondrial
	411057	*cyp314a1*	Cytochrome P450 314A1, transcript variant X6
	411893	*cyp315a1*	Cytochrome P450 315a1, mitochondrial, transcript variant X2
	724860	*cyp301b1*	Probable cytochrome P450 301a1, mitochondrial, transcript variant X1
Subcellular transportation	411685	*abcd1*	ATP-binding cassette sub-family D member 1
	552495	*abcd3*	ATP-binding cassette sub-family D member 3
	550695	*cpt1*	Carnitine O-palmitoyltransferase 1, liver isoform
	411473	*cpt2*	Carnitine O-palmitoyltransferase 2, mitochondrial-like
	411272	*acbp*	Putative acyl-CoA-binding protein
	408689	*fabp*	Fatty acid binding protein, transcript variant X2
Fatty acid degradation in peroxisome	552757	*acox1*	Probable peroxisomal acyl-coenzyme A oxidase 1
	412020	*acox3*	Peroxisomal acyl-coenzyme A oxidase 3-like, transcript variant X3
	409986	*mfe*	Peroxisomal multifunctional enzyme type 2-like, transcript variant X1
	408904	*scp*	Non-specific lipid-transfer protein
Fatty acid degradation in mitochondrial	409712	*acadsb*	Short/branched chain specific acyl-CoA dehydrogenase, mitochondrial
	411697	*acads*	Short-chain specific acyl-CoA dehydrogenase, mitochondrial
	408567	*acadm*	Probable medium-chain specific acyl-CoA dehydrogenase, mitochondrial
	412025	*acadv*	Very long-chain specific acyl-CoA dehydrogenase, mitochondrial
	409150	*e* *chs1*	Enoyl coenzyme A hydratase, short chain, 1, mitochondrial
	410325	*hadha*	Trifunctional enzyme subunit alpha, mitochondrial
	551775	*hadhb*	Trifunctional enzyme subunit beta, mitochondrial
	410325	*hadha*	Trifunctional enzyme subunit alpha, mitochondrial
	551775	*hadhb*	Trifunctional enzyme subunit beta, mitochondrial
	408291	*kat*	3-Ketoacyl-CoA thiolase, mitochondrial
	551395	*acat2*	Acetyl-CoA acetyltransferase, cytosolic
	726218	*acat1*	Acetyl-CoA acetyltransferase, mitochondrial
Fatty acid unsaturation	100576797	*desata2_* *c*	Acyl-CoA Delta(11) desaturase, transcript variant X1
	102654211	*desatb*	Acyl-CoA Delta(11) desaturase-like, transcript variant X3
	107965749	*loc107965749*	Acyl-CoA Delta(11) desaturase-like
	113218558	*loc113218558*	Acyl-CoA Delta(11) desaturase-like
	412166	*desata1*	Acyl-CoA Delta(11) desaturase
	551527	*d11ds*	Acyl-CoA Delta(11) desaturase-like, transcript variant X4
	552417	*desara2_* *a*	Acyl-CoA Delta(11) desaturase
	724226	*loc724226*	Acyl-CoA Delta(11) desaturase-like
	727166	*desate_* *b*	Acyl-CoA Delta(11) desaturase-like
	727333	*desate_* *a*	Acyl-CoA Delta(11) desaturase-like

**Table 2:** List of 10-HDA metabolism-related genes with consistent expression trends in *A. mellifera* and *A. cerana*.

Fatty Acid Process	Gene ID	Gene Symbol
Fatty acid biosynthesis	412815	*fasn*
Fatty acid biosynthesis	411662	*mecr*
Fatty acid elongation	412541	*acsl6*
Fatty acid unsaturation	551527	*d* *11ds*
Fatty acid hydroxylation	724175	*cyp342a1*
Fatty acid hydroxylation	724946	*cyp6as11*
Fatty acid hydroxylation	726690	*cyp6as3*
Fatty acid hydroxylation	551560	*cyp6bd1*
Fatty acid hydroxylation	552418	*cyp6be1*
Fatty acid hydroxylation	724860	*cyp301b1*
Fatty acid hydroxylation	551223	*cyp305d1*
Fatty acid degradation in mitochondrion	410325	*hadha*
Fatty acid degradation in mitochondrion	551775	*hadhb*
Fatty acid degradation in mitochondrion	408291	*kat*
Fatty acid degradation in mitochondrion	409150	*e* *chs* *1*
Fatty acid degradation in mitochondrion	408567	*acadm*

## Data Availability

The raw sequence data reported in this paper have been deposited in the Genome Sequence Archive (Genomics, Proteomics & Bioinformatics 2021) at the National Genomics Data Center (Nucleic Acids Res 2024), China National Center for Bioinformation/Beijing Institute of Genomics, Chinese Academy of Sciences (GSA: CRA023767 for *A. cerana* and CRA023762 for *A. mellifera*), which are publicly accessible at https://ngdc.cncb.ac.cn/gsa (accessed on 31 March 2025).

## Supplementary Materials

The following supporting information can be downloaded at: https://www.mdpi.com/article/10.3390/insects16060563/s1, Supplementary Material: the transcriptome profile of *A. mellifera* and *A. cerana*.
