# Supplementary material for: Dissecting the Molecular Mechanism of 10-HDA Biosynthesis: Role of Acyl-CoA Delta(11) Desaturase and Transcriptional Regulators in Honeybee Mandibular Glands
Source: Insects. 2025 May 26;16(6):563. doi: 10.3390/insects16060563 (PMC12193250; doi:10.3390/insects16060563)
Supplement: Supplementary file 1 [file insects-16-00563-s001.zip › supplementary_materials.pdf]

Figure S1

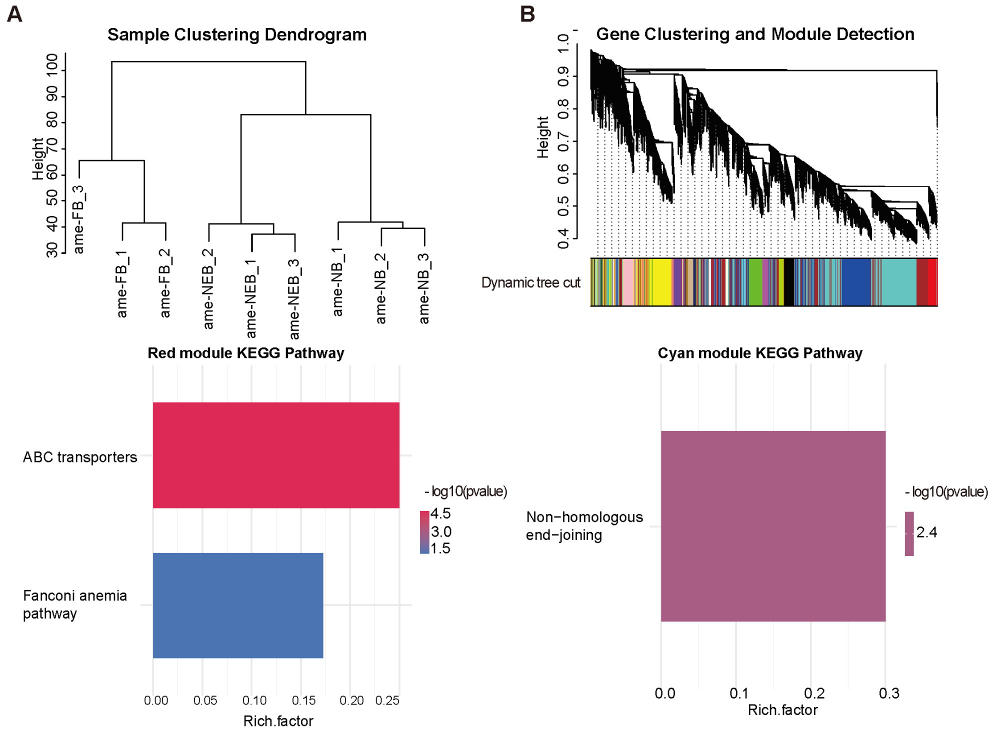

Figure S1. WGCNA analysis of the mandibular glands of NEB, NB, and FB in *Apis mellifera*. a. Hierarchical clustering dendrogram of NEB, NB and FB samples.. b. Hierarchical clustering dendrogram of co-expression modules identified by WGCNA c: KEGG pathway enrichment analysis of the genes in the red co-expression modules. d. KEGG pathway enrichment analysis of the genes in the cyan co-expression modules..

Supplementary material: The transcriptomic analysis of *Apis cerana*

Comparative analysis of *Apis cerana* mandibular glands (MGs) revealed labor-dependent transcriptional dynamics analogous to *Apis mellifera*, consistent with their shared eusocial organization [1, 2]. A total number of 9,811 genes have been identified with criteria of the gene read counts ≥ 3 in at least two biological replicates. Among these, 8,488 genes were constitutively expressed across all labor (Figure S2a). Comparative analysis identified 1,354 (NB vs NEB) and 2,827 (FB vs NEB) DEGs (|FC| ≥ 2, padj < 0.05; Figure S2b), with 29.7% (402 genes) and 52.4% (1,482 genes) upregulated in NB and FB comparisons, respectively (Figure s2b). To determine whether DEGs in the MGs of worker bees with distinct labor roles are enriched in FA-related pathways, we analyzed FA-associated genes curated from the KEGG database and GOBP terms in *Apis cerana.* the FA-related genes showed disproportionately high differential expression. While 44.4 ± 6.8% of all expressed genes were DEGs, 62.2 ± 7.56% of fatty acid-related genes exhibited labor-specific regulation (Figure S2c). To investigate the functional implications of DEGs identified in NBvsNEB and FBvsNEB comparisons within FA metabolism, we analyzed their distribution across relevant KEGG pathways and GOBP terms. KEGG pathway analysis revealed significant enrichment of DEGs in fatty acid degradation: 21 DEGs were mapped to this pathway (7 from NBvsNEB, 14 from FBvsNEB) (Figure S2d, DEGs of NB vs NEB are in grey dot bar, DEGs of FB vs NEB are in blue bar). Notably, FBvsNEB DEGs showed predominant distribution in Fatty acid elongation compared to NBvsNEB counterparts (12 DEGs in FB vs NEB, 2 DEGs in NB vs NEB). GOBP term analysis demonstrated differential distribution patterns of DEGs in key biological processes (figure S2e). 16 DEGs were mapped in Fatty acid beta-oxidation processes (5 from NBvsNEB in grey bar, 11 from FBvsNEB in blue bar), another 13 DEGs participating in general fatty acid biosynthetic process (6 from NBvsNEB, 7 from FBvsNEB). These distribution patterns suggest distinct regulatory roles of the two comparison groups in specific fatty acid metabolic processes.

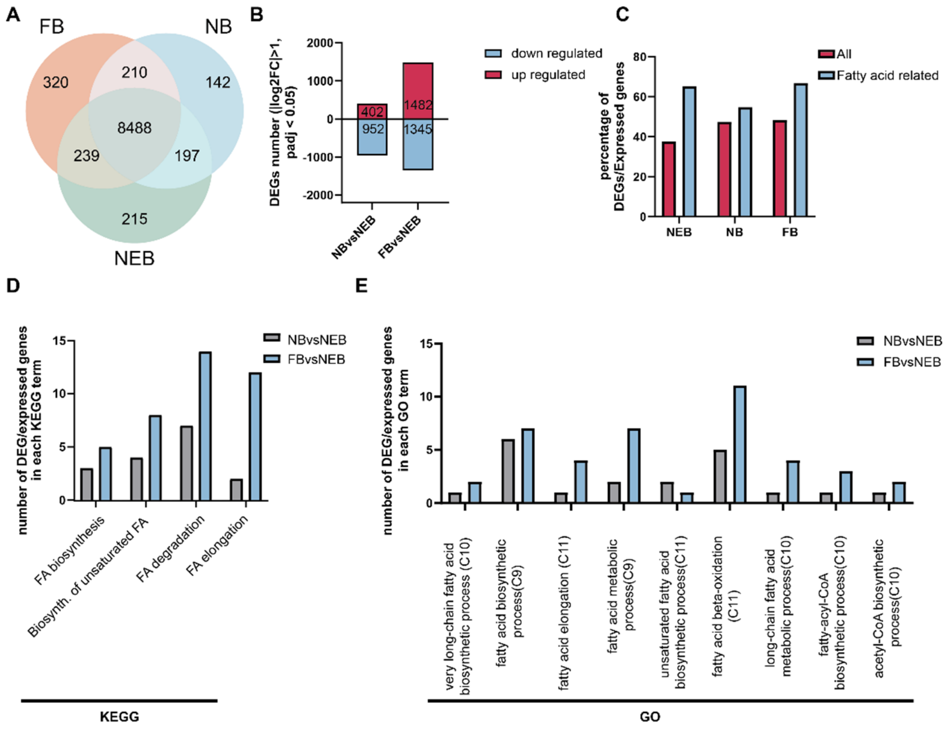

Figure S2. Functional annotation of differentially expressed genes (DEGs) in the mandibular glands of NEB, NB, and FB in *Apis cerana*. a: Distribution of expressed genes in the MGs of NEB, NB, and FB (genes with read counts ≥ 3 in at least two out of three samples are considered expressed). b: Number of up- and down-regulated DEGs in various comparisons (NB vs NEB, FB vs NEB). c: Proportion of DEGs associated with fatty acid metabolism in each KEGG term. d: Number of DEGs associated with fatty acid metabolism in each KEGG term. e: Number of DEGs associated with fatty acid metabolism in each GO term.

Reference

1. Takenaka T, Takenaka Y. Royal Jelly from Apis cerana japonica and Apis mellifera. Biosci Biotechnol Biochem. 1996;60(3):518-20.

2. Dong Z-X, Chen Y-F, Li H-Y, Tang Q-H, Guo J. The Succession of the Gut Microbiota in Insects: A Dynamic Alteration of the Gut Microbiota During the Whole Life Cycle of Honey Bees (Apis cerana). Front Microbiol. 2021;12:513962.
